# Potential Pathogenic Bacteria in Seminal Microbiota of Patients with Different Types of Dysspermatism

**DOI:** 10.1038/s41598-020-63787-x

**Published:** 2020-04-23

**Authors:** Huijun Yang, Jiaming Zhang, Zhiwei Xue, Changying Zhao, Lijun Lei, Yan Wen, Yunling Dong, Junjie Yang, Lei Zhang

**Affiliations:** 1 Maternal and Child Health Care Hospital of Shandong Province, Jinan, 250000 China; 2 Key Laboratory of Birth Regulation and Control Technology of National Health Commission of China, & Key Laboratory for Improving Birth Outcome Technique of Shandong Province, Jinan, 250000 China; 3 College of Life Science, Qilu Normal University, Jinan, 250200 China; 4 Shandong Children’s Microbiome Center, Qilu Children’s Hospital of Shandong University, Jinan, 250022 China; 5 Shandong Institute of Industrial Technology for Health Sciences and Precision Medicine, Jinan, 250100 China; 6 Shandong Institutes for Food and Drug Control, Jinan, 250101 China; 7 Qingdao Human Microbiome Center, Clinical Laboratory and Core Research Laboratory, The Affiliated Central Hospital of Qingdao University, Qingdao, 266042 China; 8 Microbiological Laboratory, Lin Yi People’s Hospital, Linyi, 276000 China; 9 Beijing Advanced Innovation Center for Big Data-Based Precision Medicine, School of Medicine and Engineering, Beihang University, & Key Laboratory of Big Data-Based Precision Medicine (Beihang University), the Ministry of Industry and Information Technology of the People’s Republic of China, Beijing, 100191 China

**Keywords:** Microbiome, Infertility

## Abstract

Human microbiota play an important role in the health of their human hosts. Recent studies have demonstrated that microbiota exist in seminal plasma. The current study aims to elucidate whether seminal microbiota exist in patients with different types of dysspermatism and whether bacterial biomarkers can be identified for them. A total of 159 study participants were recruited, including 22 patients with oligoasthenospermia, 58 patients with asthenospermia, 8 patients with azoospermia, 13 patients with oligospermia, and 58 matched healthy controls. Seminal microbiota composition was analyzed using 16S rRNA gene-based sequencing. The results showed that the composition of seminal microbiota of patients with dysspermatism differed from those of healthy controls. Comparison of the microbiota composition in semen samples from patients with different types of dysspermatism showed that microbiota in patients with asthenospermia and oligoasthenospermia were distinct from healthy controls in beta diversity (*P* < 0.05). Characteristic biomarkers, including *Ureaplasma*, *Bacteroides*, *Anaerococcus*, *Finegoldia*, *Lactobacillus* and *Acinetobacter lwoffii*, were identified based on LEfSe analysis. Inferred functional analysis based on seminal microbiome data further indicated the presence of potential pathogenic biomarkers in patients with asthenospermia and oligoasthenospermia. These results provided profiles of seminal microbiota exhibited in different types of dysspermatism, thus providing new insights into their pathogenesis.

## Introduction

Human microbiota, with its diverse relationships—commensal, parasitic, mutualistic, and pathogenic—play an important role in human health. Recent studies have reported that microbiota exist in almost every part of human body—even in the endocrine niche, such as in tumors, blood, and synovial fluid^1–4^. Advances in technology and new research have demonstrated that microbiota are found in seminal plasma, and play an important role in host homeostasis^5^. It has been demonstrated that the presence of bacteria in sperm is associated with male infertility^6^. Some bacteria in the urogenital tract may affect spermatogenesis and decrease sperm quality through various means, including decrease in sperm motility, deficiency in DNA integrity, and destruction of mitochondrial function^7^. *Escherichia coli*, *Mycoplasma genitalium*, *Ureaplasma urealyticum*, *Mycoplasma hominis*, *Staphylococcus aureus*, and *Chlamydia trachomatis* are pathogens associated with male infertility^8–12^. Several studies using high-throughput sequencing have demonstrated that seminal plasma has a bacterial community, which includes *Lactobacillus*, *Pseudomonas*, *Prevotella*, and *Gardnerella*, among others^13–19^.

Approximately 15% of couples worldwide are unable to conceive due to infertility and males contribute to 50% of the infertility cases^20,21^. There are several causative factors for male infertility, including genetic and environmental factors^21–24^. Abnormal semen (dysspermatism) is a reason for infertility, which occurs in about 50% of the cases of male infertity^25,26^. The changes in semen microenvironment could affect the spermatogenesis and motility. Many substances have recently been found in the seminal plasma that affect fertility, such as proteins, metabolites, environmental metals, etc. Compared to healthy controls, caspase-3 and cytochrome C levels were higher, and the total antioxidant capacity (TAC) was lower in seminal plasma of infertile patients^27,28^. Testosterone and androstenedione vectors correlate with steroids, which may function as biomarkers in patients with endocrine disorders, thus indicating that they may play an important role in sexual maturity^29^. A study also found that follicle-stimulating hormone deficiency could affect male fertility^30^. Several studies focused on environmental chemical substances, including perfluoroalkyl compounds, Pb, Cd, Ba, and U, have demonstrated that these substances may adversely affect seminal quality^31–33^. To summarize, seminal plasma functions not only as a medium to carry, protect, and nourish sperm after ejaculation up to fertilization, but also modulates sperm functions^34^.

It is critical to identify the bacterial species composition of the microbiota in seminal plasma to better understand the etiology and pathogenesis of urogenital tract infections and their association with infertility. A study that recruited 58 patients with infertility and 19 healthy controls observed bacteria in seminal plasma by gram staining and explored the composition of microbiota. However, the authors did not discover any differences in the microbiota in seminal plasma of patients with infertility and healthy controls^13^. Another study showed that human testes have microbiota associated with idiopathic non-obstructive azoospermia^15^. Javurek *et al*. demonstrated that there is a difference in the composition of seminal microbiota in estrogen receptor-alpha knockout male mice and mice with high-fat diet, which indicates that seminal microbiota could be affected by genetic and environmental factors, thus increasing the risk of disease to the offspring^16,17^. These studies focused on the microbiota in seminal plasma, but did not find differences between patients with dysspermatism and healthy controls, hence, they remain inconclusive^13–15,19,35^.

Although previous studies have found microbiota in the seminal plasma of males with infertility, it remains unknown whether characteristic seminal microbiota exist in patients with different types of dysspermatism. This study aspires to improve the understanding of seminal microbiota and explore the potential role of microorganisms, by analyzing the seminal plasma from 159 study participants and characterizing the microbiota profile. KEGG analysis was adopted to predict potential pathways associated with dysspermatism.

## Results

**Figure 1:**
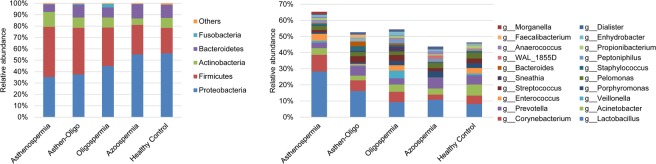
Relative abundance of taxa among five groups. Comparison of OTUs and relative taxa abundance among asthenospermia, oligospermia, oligoasthenospermia, azoospermia, and healthy controls. (**A**) At phylum level; (**B**) at genus level.

To characterize the features of seminal microbiota, 16S rRNA gene sequencing was done to measure 159 seminal samples, from 22 patients with oligoasthenospermia, 58 patients with asthenospermia, 8 patients with azoospermia, 13 patients with oligospermia, and 58 healthy controls. The clinical characteristics of the study participants are summarized in Table 1. After pre-processing of sequencing data, we obtained 3,871,353 high-quality sequences (Phred ≥ Q30) with an average of 24,348 per sample, yielding 1,065 taxa at a 97% identity cut-off. Five dominant phyla were *Proteobacteria*, *Firmicutes*, *Actinobacteria*, *Bacteroidetes* and *Fusobacteria*, as shown in Fig. 1.

**Table 1:** Characteristics of individuals investigated in this study.

	Oligoastheno-spermia	Astheno-spermia	Azoo-spermia	Oligo-spermia	Healthy control
Age (year)	33.91 ± 6.05	31.66 ± 5.97	31.25 ± 5.17	31.15 ± 5.10	30.96 ± 5.11
Sperm density (/ml)	8.66 ± 7.65	52.07 ± 39.99	N/A	13.60 ± 10.46	68.18 ± 40.50
Sperm count	59.83 ± 112.45	190.47 ± 126.27	N/A	66.84 ± 42.68	228.46 ± 118.63
pH	7.28 ± 0.14	7.27 ± 0.18	N/A	7.31 ± 0.19	7.31 ± 0.15
PR^a^	12.50 ± 7.77	20.63 ± 9.76	N/A	22.60 ± 13.29	45.88 ± 13.05
NP^b^	10.79 ± 8.79	15.75 ± 6.99	N/A	12.64 ± 6.51	19.70 ± 6.42
IM^c^	72.31 ± 20.76	63.74 ± 14.29	N/A	65.81 ± 18.84	N/A

^a^Forward motile sperm. ^b^Non-forward motile sperm. ^c^Immobile sperm.

### Altered seminal microbiota in patients with dysspermatism

**Figure 2:**
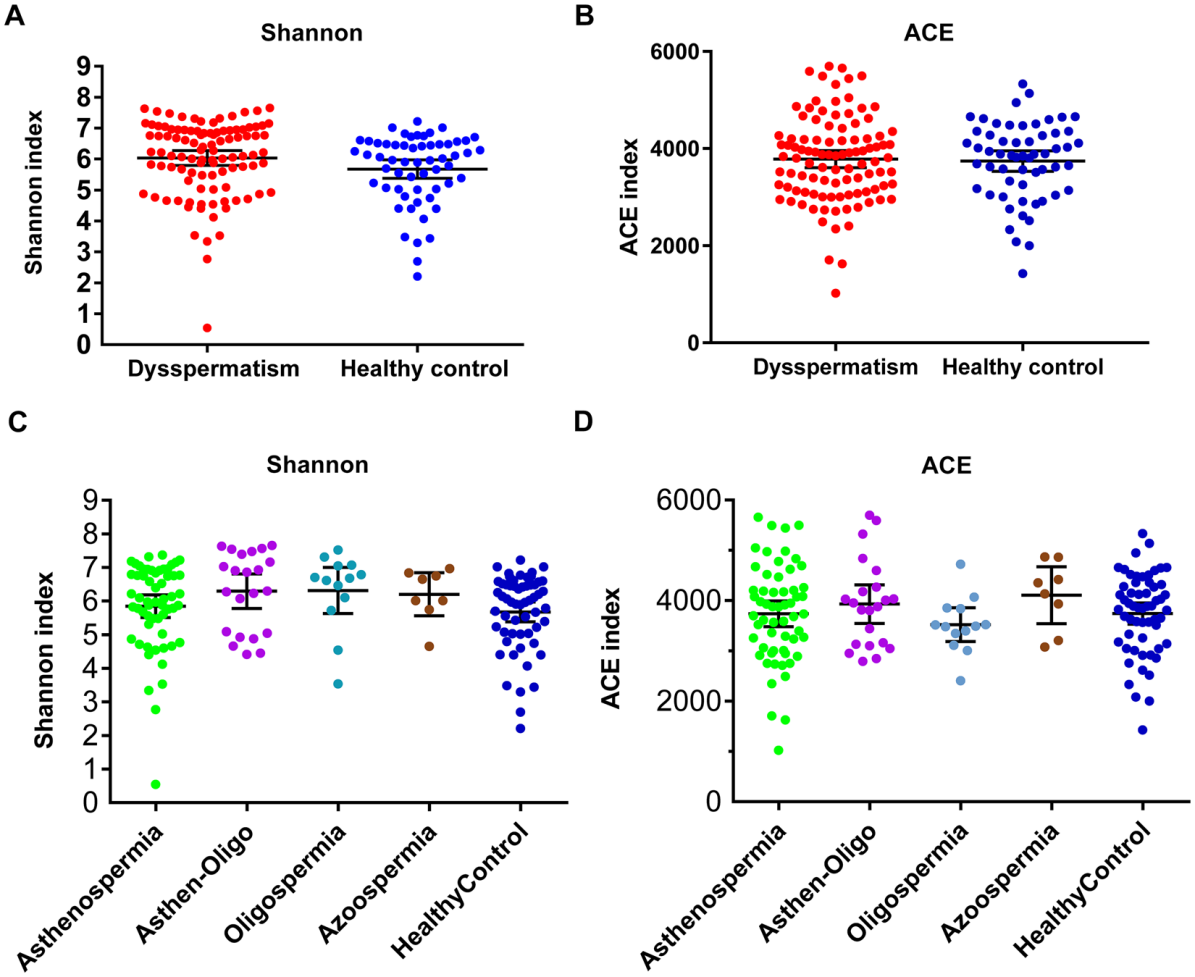
Comparison of alpha diversity and relative abundance at phylum level based on the OTUs profile. Box plots depict differences in microbiome diversity based on (**A**) Shannon index and (**B**) ACE index between patients with dysspermatism and healthy controls. (**C**,**D**) show Shannon index and ACE index among patients with asthenospermia, oligospermia, oligoasthenospermia, and azoospermia, and healthy controls. The p value was calculated using the Wilcoxon rank-sum test.

**Figure 3:**
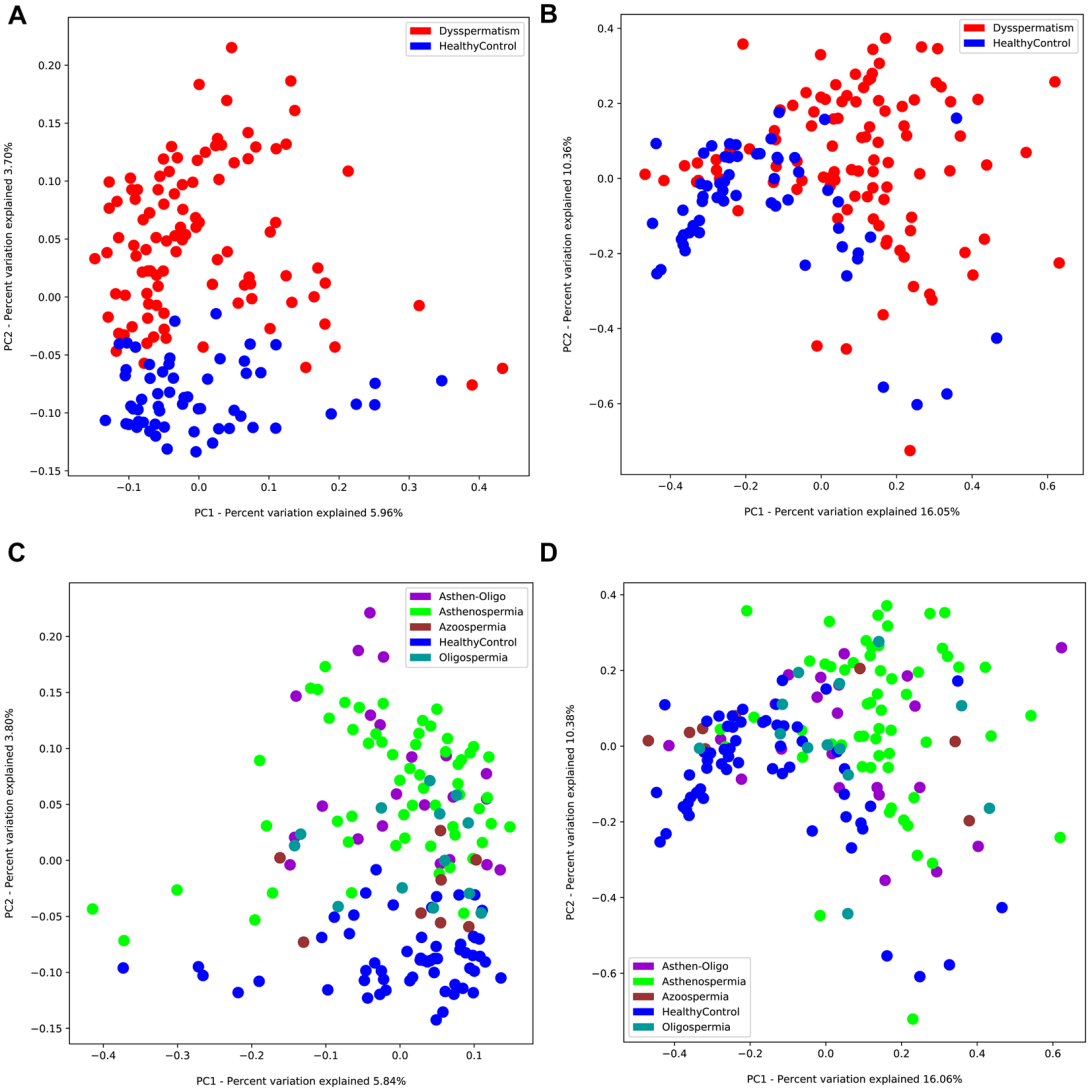
PCoA analysis of microbiota between patients with dysspermatism and healthy controls. (**A**) Unweighted unifrac PCoA; (**B**) Weighted unifrac PCoA. PCoA analysis of the microbiota among patients with asthenospermia, oligospermia, oligoasthenospermia, and azoospermia, and healthy controls; (**C**) Unweighted unifrac PCoA; (**D**) Weighted unifrac PCoA.

**Figure 4:**
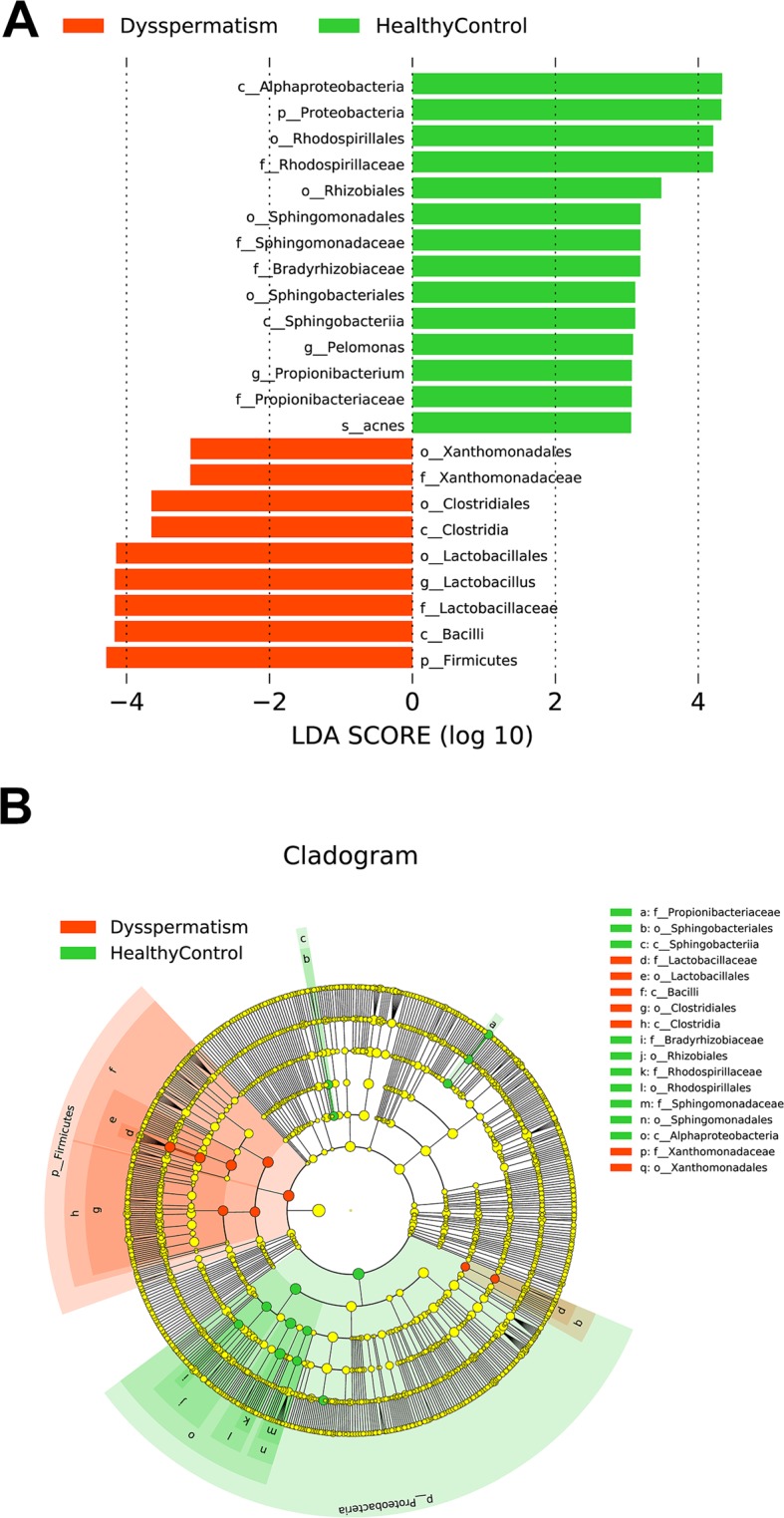
The most differentially abundant taxa between patients with dysspermatism and healthy controls (LDA score above 3) generated using LEfSe analysis.

The aim of the analysis of seminal microbiota was to better understand the differences in seminal microbiota between patients with dysspermatism and healthy controls. Although α diversity including ACE index (*P* > 0.05) and Shannon index (*P* > 0.05) showed no significant difference in patients with dysspermatism and the healthy control group (Fig. 2A,B), β diversity (based on the unweighted and weighted UniFrac distance) was significantly different between the two groups (r = 0.598, *P* = 0.001 weighted UniFrac; r = 0.972, *P* = 0.001, unweighted UniFrac, Fig. 3A,B). A total of 110 significantly different taxa were detected in the two groups. Seminal microbiota in the dysspermatism group was characterized by dominance of genera of *Lactobacillus*, *Bacteroides*, *Delftia*, *Sneathia*, *Enhydrobacter*, *Anaerococcus*, *Mycoplana*, *Finegoldia*, *Stenotrophomonas*, *Methylobacterium*, *Coprobacillus*, *Aerococcus*, *Atopobium*, *Chryseobacterium*, *Kocuria*, *Megasphaera*, *Ralstonia*, *Achromobacter*, *Erwinia*, *Ureaplasma*, and *Filifactor*, and species of *Prevotella copri*, *Saccharopolyspora hirsuta*, *Kocuria palustris*, *Prevotella nigrescens*, *Porphyromonas endodontalis*, *Lactobacillus coleohominis*, *Bacteroides barnesiae*, and *Lactobacillus iners*. On the other hand, the microbiota in the healthy control group was dominated by genera of *Pelomonas*, *Propionibacterium*, *Bosea genosp*, *Bosea*, *Afipia*, *Sphingomonas*, *Vogesella*, *Brevibacillus*, *Xylanimicrobium*, *Flexispira*, *Pedomicrobium*, *Phyllobacterium*, *Aquimonas*, *Dietzia*, *Sediminibacterium*, *Mycobacterium*, and *Eikenella*, and species of *Brevibacterium aureum*, *Propionibacterium acnes*, *Corynebacterium simulans*, *Eubacterium dolichum*, and *Bacillus thermoamylovorans* (*P* < 0.05, Figs. 4 and S1A). In summary, we found that the composition of seminal microbiota was different between the two groups.

### Patients with asthenospermia or oligoasthenospermia harbor an altered seminal microbiota compared to healthy controls

There are differences in the specific pathogenesis of different types of dysspermatism. To determine the role of seminal microbiota in the different types of dysspermatism, we analyzed the seminal microbiota in patients with four different types of dysspermatism—asthenospermia, oligospermia, oligoasthenospermia, and azoospermia, and compared to healthy controls. There was no significant difference in α diversity (Shannon index and ACE index) among the five groups (*P* > 0.05, Fig. 2C,D). Analysis of β diversity showed seminal microbiota of patients with oligospermia (r = 0.180, *P* = 0.044, weighted UniFrac; r = 0.105, *P* = 0.110, unweighted UniFrac) or azoospermia (r = 0.207, *P* = 0.073, weighted UniFrac; r = 0.169, *P* = 0.096, unweighted UniFrac) showed no significant difference when compared to that of healthy controls; whereas seminal microbiota in patients with asthenospermia (r = 0.294, *P* = 0.0001, weighted UniFrac; r = 0.362, *P* = 0.0001, unweighted UniFrac) or oligoasthenospermia (r = 0.270, *P* = 0.001, weighted UniFrac; r = 0.316, *P* = 0.001, unweighted UniFrac) had significant composition variations compared to that of healthy controls (Fig. 3C,D).

### Patients with asthenospermia harbored unique bacterial biomarkers, which may have potential pathogenicity

**Figure 5:**
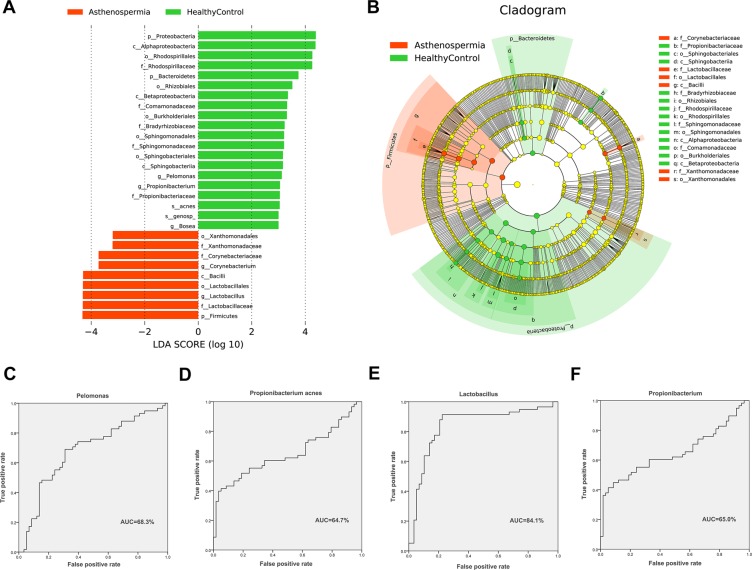
LEfSe analysis between patients asthenospermia and healthy controls. (**A**,**B**) Comparison of the most differentially abundant taxa between patients with asthenospermia and healthy controls (LDA score above 3) generated using LEfSe analysis. We selected 4 biomarkers to predict the probability of patients with asthenospermia. (**C**–**F**) These biomarkers are *Propionibacterium*, *Pelomonas*, *Lactobacillus*, and *Propionibacterium acnes*. The ROC curves as well as the AUC (Area Under the Curve) values were calculated using SPSS.

LEfSe analysis (Linear discriminant analysis Effect Size) was used to explore the bacterial biomarkers in the semen of patients with asthenospermia. Eighty different taxa in the two groups were chosen based on LDA > 2. Significant increase in the relative abundance of *Sneathia*, *Ralstonia*, *Ureaplasma*, *Bacteroides*, *Chryseobacterium*, *Aerococcus*, *Enhydrobacter*, *Methylobacterium*, *Anaerococcus*, *Stenotrophomonas*, *Mycoplana*, *Delftia*, *Finegoldia*, *Corynebacterium*, and *Lactobacillus* (at the genus level) and *Saccharopolyspora hirsute*, *Acinetobacter lwoffii*, and *Lactobacillus iners* (at the species level) was observed, and a significant reduction in *Pelomonas*, *Propionibacterium*, *Bosea*, *Sphingomonas*, *Phyllobacterium*, *Pedomicrobium*, *Xylanimicrobium*, *Mycobacterium*, and *Zoogloea* (at the genus level) and *Propionibacterium acnes* and *Bosea genosp* (at the species level) was observed in the asthenospermia group, compared to the healthy control group (Figs. 5A,B and S1B).

To evaluate the potential value of the identified bacterial biomarkers for asthenospermia, ROC curves and AUC values were computed. The criteria used for biomarkers are—the genus and species of LDA > 3. Four biomarkers including *Propionibacterium* (ROC-plot AUC value was 0.650, 95% confidence interval [CI]: 54.6–75.3%), *Pelomonas* (ROC-plot AUC value was 0.683, 95% CI: 58.4–78.2%), *Lactobacillus* (ROC-plot AUC value was 0.841, 95% CI: 76.2–92.0%), and *Propionibacterium acnes* (ROC-plot AUC value was 0.647, 95% CI: 54.4–75.1%) were filtered. Additionally, we evaluated the effects of age and pH of seminal plasma on the four candidate biomarkers, in patients with asthenospermia and those in healthy controls (Fig. 5C–F). None of these factors had significant effect on the selected candidate biomarkers (Table S1).

### Unique pathogenic bacteria in patients with oligoasthenospermia

**Figure 6:**
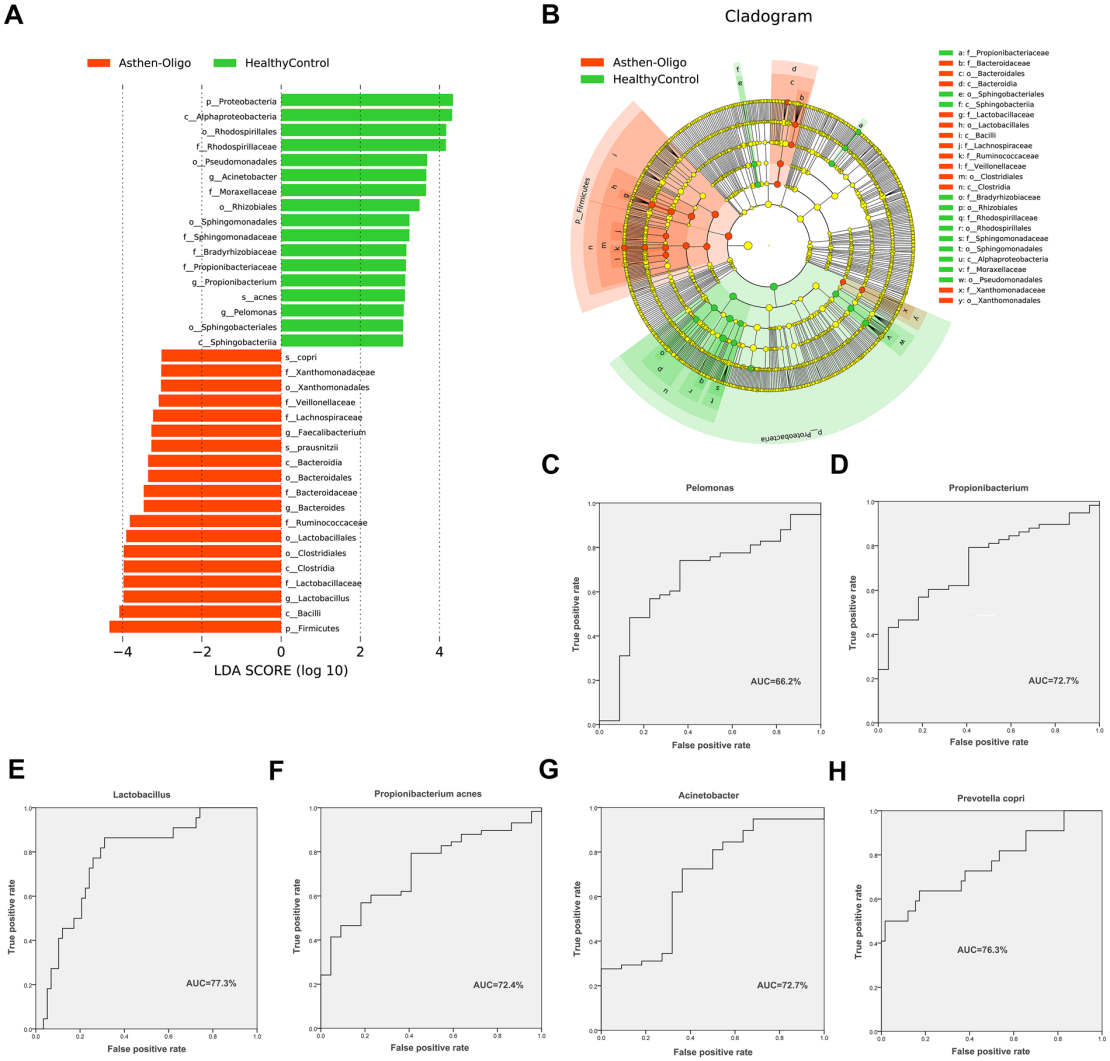
LEfSe analysis between patients with oligoasthenospermia and healthy controls. (**A**,**B**) Comparison of the most differentially abundant taxa between patients with oligoasthenospermia and healthy controls (LDA score above 3), were generated using LEfSe analysis. (**C**–**H**) These biomarkers are *Lactobacillus*, *Acinetobacter*, *Propionibacterium*, *Pelomonas*, *Prevotella copri*, and *Propionibacterium acnes*. The ROC curves as well as the AUC (Area Under the Curve) value was calculated using SPSS.

In order to select the biomarkers in oligoasthenospermia and healthy controls, we used LEfSe to analyze the composition of seminal microbiota in both groups. Seminal microbiota in the oligoasthenospermia group were characterized by a dominance of *Ralstonia*, *Oscillospira*, *Parabacteroides*, *Lachnospira*, *Phascolarctobacterium*, *Chryseobacterium*, *Zoogloea*, *Ruminococcus*, *Stenotrophomonas*, *Actinoplanes*, *Mycoplana*, *Delftia*, *Sneathia*, *Megasphaera*, *Atopobium*, *Faecalibacterium*, *Bacteroides*, *Lactobacillus*, *Bacteroides uniformis*, *Stenotrophomonas panacihumi*, *Bacteroides plebeius*, *Prevotella copri*, and *Faecalibacterium prausnitzii*, whereas microbiota in healthy controls were dominated by *Acinetobacter*, *Propionibacterium*, *Pelomonas*, *Bosea*, *Deinococcus*, *Sphingomonas*, *Sediminibacterium*, *Pseudomonas*, *Pedomicrobium*, *Acinetobacter johnsonii*, *Streptococcus anginosus*, *Propionibacterium acnes*, and *Bosea genosp* (Figs. 6A,B and S1C).

As previously described, 6 biomarkers were selected in patients with oligoasthenospermia and healthy controls, which contained *Lactobacillus* (ROC-plot AUC value was 0.773, 95% CI: 66.2–88.4%), *Acinetobacter* (ROC-plot AUC value was 0.679, 95% CI: 54.5–81.3%), *Propionibacterium* (ROC-plot AUC value was 0.727, 95% CI: 61.3–84.1%), *Pelomonas* (ROC-plot AUC value was 0.662, 95% CI: 52.9–79.6%), *Prevotella copri* (ROC-plot AUC value was 0.763, 95% CI: 63.2–89.3%), and *Propionibacterium acnes* (ROC-plot AUC value was 0.724, 95% CI: 61.0–83.9%) (Fig. 6C–H). We also evaluated these candidate biomarkers by using confounding factors and found that there were no confounding factors that could affect these biomarkers (Table S2).

### Predicted metagenome functions in patients with asthenospermia and healthy controls, and patients with oligoasthenospermia and healthy controls

**Figure 7:**
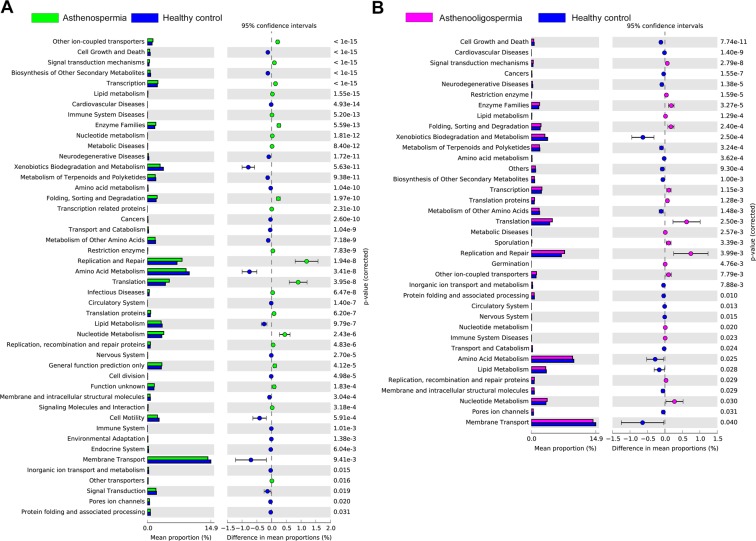
Predicted metagenome function based on KEGG pathway analysis. Extended error bar plot showed significantly different KEGG pathways between (**A**) patients with asthenospermia and healthy controls; (**B**) between patients with oligoasthenospermia and healthy controls.

Phylogenetic Investigation of Communities by Reconstruction of Unobserved States (PICRUSt) was used to predict the different KEGG pathways in the two groups, and to discuss potential mechanisms of seminal microbiota in asthenospermia and oligoasthenospermia groups. Forty different KEGG pathways were displayed in patients with asthenospermia; these exhibited increased activities in some disease pathways such as cell growth and death, lipid metabolism, enzyme families, infectious diseases, cell division, cell motility, etc. There are 32 different KEGG pathways in patients with oligoasthenospermia and healthy controls, which showed increased pathways in cell growth and death, lipid metabolism, and metabolic diseases in patients with oligoasthenospermia (Fig. 7).

## Discussion

In this study, high-throughput sequencing technology was used to analyze and measure the microbiota found in seminal plasma. Our study demonstrated that patients with different types of dysspermatism (oligoasthenospermia or asthenospermia) had significantly different composition of microbiota when compared to healthy controls. This pilot study also explored the opportunistic pathogens in the seminal plasma, which could be potential pathogens that increase the risk of dysspermatism.

Technological advances in recent years have led to several studies on the relationship between microbiota and human health. A large number of studies have shown that disorders of human microbial groups can lead to diseases, such as type II diabetes^36^. Recent studies have shown that bacteria also exist in the synovial fluid of patients with arthritis and play an important role in the occurrence of arthritis^3^. More interestingly, there are microorganisms in the blood^4^. This evidence indicates that different niches of the human body environment harbor distinct and potentially functional microbiota. In recent years, high throughput sequencing has been used to measure bacteria in seminal plasma, by which *Lactobacillus*, *Pseudomonas*, *Prevotella Streptococcus*, and *Gardnerella* were revealed to exist in seminal samples of patients and healthy controls^13–15,19,35^. These studies also found potentially pathogenic bacteria, such as *Finegoldia* and *Anaerococcus*^13,14^. We analyzed the composition of microbiota in the seminal plasma at the phylum level and found five dominant phyla: Proteobacteria, Firmicutes, Actinobacteria, Bacteroidetes, and Fusobacteria, which is consistent with the findings of a previous study^15^. We also analyzed major genera in the seminal plasma. The top 10 relatively abundant genera found commonly in the male seminal plasma were: *Lactobacillus*, *Corynebacterium*, *Acinetobacter*, *Prevotella*, *Enterococcus*, *Veillonella*, *Streptococcus*, *Porphyromonas*, *Sneathia*, and *Pelomonas*^37^. Based on all current studies, microbiota exist in the seminal plasma and share similar microbial community composition^13–18,35^.

Variation in the composition of microbiota could affect patients with asthenospermia or oligoasthenospermia. We found most of the taxa that were found in increased concentration in the seminal fluid of patients with asthenospermia and oligoasthenospermia were gram-negative bacteria containing lipopolysaccharide (LPS) in their cell walls. LPS can upregulate cytokines causing inflammation^38^. A previous study indicated that inflammatory mediators can directly cause DNA fragmentation in ejaculated spermatozoa, which ultimately limits the fertilization abilities of the germ cells^39^.

The relative abundance of *Ureaplasma*, *Bacteroides*, *Anaerococcus*, *Finegoldia*, *Lactobacillus*, and *Acinetobacter lwoffii* was significantly higher in asthenospermia, and *Lactobacillus* was notably abundant in oligoasthenospermia. *Ureaplasma* is the smallest prokaryote between bacteria and viruses, mainly found in the genitourinary tract of the human body. *Bacteroides ureolyticus* is a species of *Bacteroides* that could impair sperm structure and function by diminishing motility and causing sperm membrane injury, especially to the lipid bilayers as shown in *in vitro* tests^29,40,41^. Another study using 16S rRNA sequencing demonstrated that genus *Anaerococcus* could be a biomarker for predicting male infertility^13^. We also found this bacterium in patients with asthenospermia. *Lactobacillus* is a gram-positive bacterium, which produces SCFA (short-chain fatty acids). Recent studies have shown that SCFA are beneficial to human health. However, seminal pH is generally 7.2^42^. Significant increase of *Lactobacillus* in asthenospermia may change the pH of semen and result in male infertility. Although our results found *Lactobacillus* in human semen, the mechanism remains to be determined, however, it provides new insights into male infertility.

Bacteria that were filtered out by factors such as age may be potentially pathogenic. The characteristic of asthenospermia is that the activity of spermatozoa is weakened. We found an increase in *Lactobacillus* and decrease in *Propionibacterium*, *Pelomonas*, and *Propionibacterium acnes* in the seminal plasma of patients with asthenospermia, which indicated that these bacteria may affect sperm activity. Oligoasthenospermia features both lesser and weaker sperm activity. Our results indicated an increase in *Lactobacillus* and *Prevotella copri* and decrease in *Propionibacterium*, *Pelomonas*, *Acinetobacter*, and *Propionibacterium acnes*, which may correlate with sperm formation and activity.

PICRUSt was used to predict potential metabolic pathways. We found that the lipid metabolism was significantly different in the three experimental groups. The essence of sex hormones is steroids. Previous studies have demonstrated that disorders of sex hormones result in male infertility. For instance, changes in FSH, LH, and T levels are associated with damage to the testes, impeding of spermatogenesis and maturation, and result in decreased sperm motility and activity, leading to infertility^43–45^. This finding also exhibits strong correlation with the change in microbiota.

To summarize, our results suggest that dysspermatism is associated with seminal microbiota, and also show that seminal microbiota in patients with asthenospermia and oligoasthenospermia are different from those in healthy controls. Biomarkers were screened and the KEGG metabolic pathways predicted. These results are beneficial for clinical diagnosis and could be further used to develop new treatment for patients with dysspermatism. Although we performed a bacterial culture experiment on semen samples, the pathogens were not cultured because of their low content. To better understand the mechanism of dysspermatism, future studies are needed in combination with metabolomics and culturomics.

## Methods

### Study participants

A total of 159 study participants from Shandong Health Center for Women & Children were recruited, including 22 patients with oligoasthenospermia (having both characteristics of asthenospermia and oligospermia), 58 patients with asthenospermia (the total number of sperm less than 39 × 10^6^/ml), 8 patients with azoospermia (the absence of sperm or very low sperm content in semen), 13 patients with oligospermia (the proportion of progressive motility is less than 32%), and 58 healthy controls^42^. The study participants were about 31.65 ± 6.01 years old, and there was no significant difference among these groups. The diagnostic criteria of dysspermatism followed the 5^th^ WHO laboratory manual for examination and processing of human semen^42^. Signed informed consent was taken and clinical indexes were also collected. All study participants met the following criteria: (1) study participants hadn’t taken antibiotics for three months prior to study enrollment, (2) study participants and their family members have no known genetic disease, (3) no history of sexually transmitted diseases, and (4) no history of corticosteroid use. All study procedures were approved by the Medical Ethical Committee of Maternal and Child Health Care Hospital of Shandong Province (#IRB: 2017-03). All methods and experimental protocols in this study were performed in accordance with relevant guidelines and standard operating procedures.

### Collection of seminal plasma samples

In order to prevent contamination, stringent criteria and procedures were adopted to collect seminal plasma samples by masturbation. All participants abstained from sexual activity for 3 to 7 days before sample collection. Before sampling, hands were washed thoroughly with soap 2 to 3 times. The penis, especially the glans and coronal sulcus, was washed with warm soapy water and then wiped 2 to 3 times with 75% alcohol. The seminal plasma was injected directly into sterile glass containers, avoiding skin contact with the interior wall of the container. Fresh semen was used for routine seminal plasma clinical testing, and the remaining seminal samples were transferred to sterile microcentrifuge tubes and stored at −80 °C, within 2 hours of collection.

### Isolation of seminal fluid microbial DNA and 16S rRNA sequencing

About 400 μl of seminal specimens were used for genomic DNA extraction, which was extracted using the CTAB (Cetyl trimethylammonium bromide) method^46^. Nanodrop 2000 (Thermo Scientific) spectrophotometer was used to determine the concentration of extracted DNA. The V1–V2 regions of 16S rRNA gene were amplified and sequenced on an Illumina HiSeq. 2500 system. PCR was conducted using bacterial universal primers 27F (5′ -(6FAM) AGA GTT TGA TCC TGG CTC AG 3′) and 355R (5′ GCT GCC TCC CGT AGG AGT 3′). Each PCR reaction contained 12.5 ul Q5 Hot Start High-Fidelity 2X Master Mix (BioLabs), 1.25 μl 10 μM Forward Primer, 1.25 ul 10 μM Reverse Primer, and 10 μl DNA template in a total volume of 25 μl. The following experiments were carried out as per the sequencing manual.

### rRNA sequencing analysis

The 16S sequence paired-end data set was joined and qualitatively filtered using the Laser FLASH method described by Magoč and Salzberg^47^. All sequences were analyzed using the Quantitative Insights into Microbial Ecology (QIIME, version 1.9.1) software suite^48^, according to the QIIME tutorial (http://qiime.org/) with a few modified methods. Chimeric sequences, where a single organism has distinct genotypes, were removed using Metagenomics tool—usearch61 with denovo models^49^. Sequences were clustered against the 2013 Greengenes (13_5 release) ribosomal database’s 97% reference data set (http://greengenes.secondgenome.com/downloads). Sequences that remained unmatched with any of the entries in this reference were subsequently clustered into de novo OTUs at 97% similarity, using UCLUST algorithm. Taxonomy was assigned to all OTUs using the RDP classifier^50^ within QIIME and the Greengenes reference data set. Rarefaction and rank abundance curves were calculated from OTU tables using alpha diversity and rank abundance scripts within the QIIME pipeline. The hierarchical clustering based on population profiles of most common and abundant taxa was performed using UPGMA clustering (Unweighted Pair Group Method with Arithmetic mean, also known as average linkage) on the distance matrix of OTU abundance. This resulted in a Newick-formatted tree, obtained utilizing the QIIME package. Furthermore, QIIME was used to analyze alpha diversity (Shannon, ACE), beta diversity (weighted UniFrac, Principal Coordinate Analysis (PCoA)), Linear discriminant analysis (LDA) and Effect Size (LEfSe). SPSS (version 23) was used to calculate the receiver operating characteristic (ROC) curve and the area under the curve (AUC) values.

### Data

All sequencing data were submitted to the NCBI SRA database (accession number: PRJNA534354).

### Statistical analysis

The clinical characteristics of the study participants are represented as the mean ± SD, which were determined using Mann-Whitney U test. The diversity categorization of alpha and beta diversity is defined in the OTU table to a sequencing depth of 1,000 per sample. Moreover, alpha diversity was determined using Mann-Whitney U test and beta diversity was acquired using ANOSIM (Analysis of Similarities). LEfSe combines Kruskal-Wallis test or pair wise Wilcoxon rank sum test with linear discriminant analysis (LDA), whose threshold value on the logarithmic LDA score equals to 2.0. Analyses were performed using the SPSS statistical package (version 23). *P* values <0.05 were considered statistically significant.

### Ethical approval and informed consent

All study procedures were approved by the Medical Ethical Committee of Maternal and Child Health Care Hospital of Shandong Province (#IRB: 2017-03). All study participants signed informed consent forms and were from the same geographical area. Data was collected using a standardized questionnaire that included basic information, medical history, and examination results.

## Supplementary information

Supplementary information
