# Supplementary material for: Potential Pathogenic Bacteria in Seminal Microbiota of Patients with Different Types of Dysspermatism
Source: Sci Rep. 2020 Apr 23;10:6876. doi: 10.1038/s41598-020-63787-x (PMC7181748; doi:10.1038/s41598-020-63787-x)
Supplement: Supplementary file 1 — Supplementary information [file 41598_2020_63787_MOESM1_ESM.pdf]

Supplementary materials for Manuscript # SREP-19-23883D

Title: Potential Pathogenic Bacteria in Seminal Microbiota of Patients with Different Types of Dysspermatism

Running title: Seminal microbiota in patients with dysspermatism

Huijun Yang [1, 2], Jiaming Zhang [3, 4], Zhiwei Xue [1, 2], Changying Zhao [4, 5], Lijun Lei [1, 2], Yan Wen [1, 2], Yunling Dong [1, 2], Junjie Yang [3, 6, 7, 8, *], Lei Zhang [5, 9, *]

[1] *Maternal and Child Health Care Hospital of Shandong Province, Jinan 250000, China*

[2] *Key Laboratory of Birth Regulation and Control Technology of National Health Commission of China, & Key Laboratory for Improving Birth Outcome Technique of Shandong Province, Jinan 250000, China*

[3] *College of Life Science, Qilu Normal University, Jinan 250200, China*

[4] *Shandong Children's Microbiome Center, Qilu Children's Hospital of Shandong University, Jinan 250022, China*

[5] *Shandong Institute of Industrial Technology for Health Sciences and Precision Medicine, Jinan 250100, China*

[6] *Shandong Institutes for Food and Drug Control, Jinan 250101, China*

[7] *Qingdao Human Microbiome Center, Clinical Laboratory and Core Research Laboratory, The Affiliated Central Hospital of Qingdao University, Qingdao 266042, China*

[8] *Microbiological Laboratory, Lin Yi People's Hospital, Linyi 276000, China*

[9] *Beijing Advanced Innovation Center for Big Data-Based Precision Medicine, School of Medicine and Engineering, Beihang University, & Key Laboratory of Big Data-Based Precision Medicine (Beihang University), the Ministry of Industry and Information Technology of the People's Republic of China, Beijing 100191, China*

* Corresponding authors
Lead correspondence
Lei Zhang, Ph.D.,
Prof. of Microbiology and BME
Microbiome-X Lab
Beijing Advanced Innovation Center for Big Data-Based Precision Medicine, School of Medicine and Engineering, Beihang University;
Key Laboratory of Big Data-Based Precision Medicine (Beihang University), the Ministry of Industry and Information Technology of the People's Republic of China
Beijing 100191, CHINA
M: +86 186 1826 8987
Email: microbiome@foxmail.com.

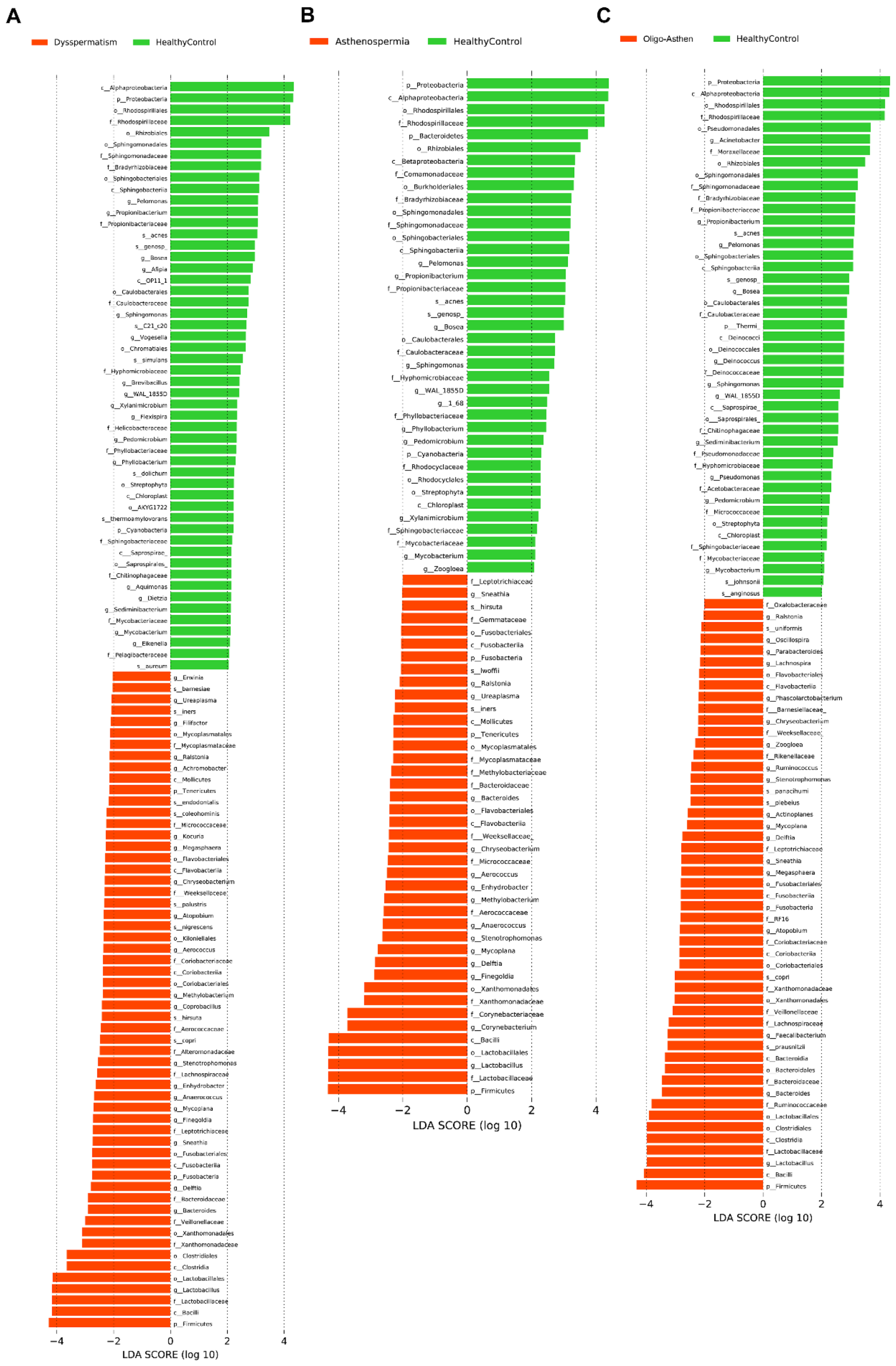

Figure S1. LEfSe analysis between (A) dysspermatism and healthy control; (B)

asthenospermia and healthy control; (C) oligoasthenospermia and healthy control based on LDA > 2.

Table S1. Effect of Age and pH on the Discovered Biomarkers for Asthenospermia vs. Healthy control

	Asthenospermia		Healthy control	
	age	pH	age	pH
Propionibacterium	0.877	0.688	0.099	0.46
Lactobacillus	0.47	0.12	0.05	0.488
Pelomonas	0.596	0.89	0.158	0.396
Propionibacterium acnes	0.88	0.699	0.1	0.467

The effects of each of the two factors, i.e. Age and pH on the validated biomarkers were examined within each of the 2 clinical categories by the SPSS one way ANOVA test. The effect was considered significant if *p-value* < 0.05.

Table S2. Effect of Age and pH on the Discovered Biomarkers for Asthenooligospermia vs. Healthy control

	Asthenooligospermia		Healthy control	
	age	pH	age	pH
Propionibacterium	0.274	0.436	0.099	0.46
Lactobacillus	0.95	0.022	0.05	0.488
Pelomonas	0.297	0.701	0.158	0.396
Acinetobacter	0.112	0.332	0.994	0.785
Propionibacterium acnes	0.272	0.441	0.1	0.467
Prevotella copri	0.184	0.841	0.17	0.143

The effects of each of the two factors, i.e. Age and pH on the validated biomarkers were examined within each of the 2 clinical categories by the SPSS one way ANOVA test. The effect was considered significant if *p-value* < 0.05.
